# Pathophysiology of Congenital High Production of IgE and Its Consequences: A Narrative Review Uncovering a Neglected Setting of Disorders

**DOI:** 10.3390/life14101329

**Published:** 2024-10-18

**Authors:** Francesca Galletta, Antonella Gambadauro, Simone Foti Randazzese, Stefano Passanisi, Vito Sinatra, Lucia Caminiti, Giuseppina Zirilli, Sara Manti

**Affiliations:** Pediatric Unit, Department of Human Pathology in Adult and Developmental Age ‘Gaetano Barresi’, University of Messina, 98124 Messina, Italy; francygall.92@gmail.com (F.G.); gambadauroa92@gmail.com (A.G.); simone.foti.92@gmail.com (S.F.R.); vitosinatra@tiscali.it (V.S.); lucia.caminiti@unime.it (L.C.); giuseppina.zirilli@unime.it (G.Z.)

**Keywords:** inborn errors of immunity, primary immunodeficiency, immunological disorders, hyper-IgE syndrome, omenn syndrome, wiskott-aldrich syndrome, IPEX syndrome

## Abstract

Elevated serum IgE levels serve as a critical marker for uncovering hidden immunological disorders, particularly inborn errors of immunity (IEIs), which are often misdiagnosed as common allergic conditions. IgE, while typically associated with allergic diseases, plays a significant role in immune defense, especially against parasitic infections. However, extremely high levels of IgE can indicate more severe conditions, such as Hyper-IgE syndromes (HIES) and disorders with similar features, including Omenn syndrome, Wiskott-Aldrich syndrome, and IPEX syndrome. Novel insights into the genetic mutations responsible for these conditions highlight their impact on immune regulation and the resulting clinical features, including recurrent infections, eczema, and elevated IgE. This narrative review uniquely integrates recent advances in the genetic understanding of IEIs and discusses how these findings impact both diagnosis and treatment. Additionally, emerging therapeutic strategies, such as hematopoietic stem cell transplantation (HSCT) and gene therapies, are explored, underscoring the potential for personalized treatment approaches. Emphasizing the need for precise diagnosis and tailored interventions aims to enhance patient outcomes and improve the quality of care for those with elevated IgE levels and associated immunological disorders.

## 1. Introduction

Immunoglobulin E (IgE) was discovered in 1966 by Kimishige Ishizaka and his wife, Teruko Ishizaka [1,2]. They isolated this new “reagin” from the serum of a person with a high sensitivity to ragweed pollen and called this new class of immunoglobulin “erythema (E)-induced gamma globulin” [1,2]. Despite its lower concentration in the serum of healthy people compared to other immunoglobulins (i.e., IgA, IgG, IgM), IgE exerts extremely rapid actions on a wide range of several cells through its receptors, named high-affinity Fc receptor for IgE (FcεRI) and low-affinity Fc receptor for IgE (FcεRII, or CD23) [3,4]. The cellular effectors include mast cells, basophils, dendritic cells (DCs), eosinophils, Langerhans cells, macrophages, monocytes, neutrophils, and B cells [3]. IgE is implicated in host defense (especially against parasitic infections) and immune regulation [5,6]. It is responsible for type I hypersensitivity reactions (e.g., anaphylaxis) and humoral memory immune responses, and it may also be implicated in anti-tumor immunity [7,8]. The normal range of serum IgE is ~2–214 IU/mL, which is 10,000 to 50,000-fold less than IgG [9,10]. Increased IgE levels are defined by two standard deviations (SD) above the mean, but when the levels exceed 2000 IU/mL, they are considered markedly elevated or “hyper IgE” [11,12,13]. In most countries with low frequency of parasitic infections, high serum IgE levels are more commonly associated with atopic diseases, such as allergic asthma, atopic dermatitis, allergic rhinitis with or without conjunctivitis, and food allergy [14,15]. However, some conditions can be related to inborn errors of immunity (IEIs), also known as primary immunodeficiency disorders (PIDs), characterized by different combinations of severe and/or recurrent infections, allergy, autoimmunity, autoinflammatory diseases, bone marrow failure, and/or malignant manifestations [16]. These conditions represent a growing group of diseases, and patients affected by IEIs constitute a significant health burden [16,17]. Accurately estimating the prevalence of IEI remains challenging due to underdiagnosis and the variability of genetic and environmental factors across different populations [16]. IEIs are mostly monogenic disorders related to mutations in genes responsible for immune regulation and host defense, and affected individuals often preset the allergic triad characterized by high IgE levels, eosinophilia, and eczema [18,19]. The distinction between common allergic diseases and IEIs in individuals with elevated serum IgE levels can be difficult, leading to misdiagnosis [20]. IEIs predominantly associated with increased IgE levels can be classified in the following: Hyper-IgE syndromes (HIES), and other conditions that can mimic HIES, such as Omenn syndrome (OS), Wiskott-Aldrich syndrome (WAS), and immune dysregulation, polyendocrinopathy, enteropathy, and X-linked (IPEX) [16,17,18]. The purpose of this review was to explore the relationship between high serum IgE levels and underlying IEIs. A comprehensive overview of the existing literature, based on a critical evaluation without standardized methodologies or statistical analyses, was carried out using the PubMed database. Key terms included “IgE” OR “Immunoglobulin E” AND “inborn errors of immunity” OR “IEI” OR “primary immunodeficiency disorders”. Randomized controlled studies, observational studies, meta-analyses, reviews, and evidence-based guidelines were included in our analysis.

## 2. Pathophysiology of Elevated IgE Level

### 2.1. Regulation of IgE Production

**Figure 1:**
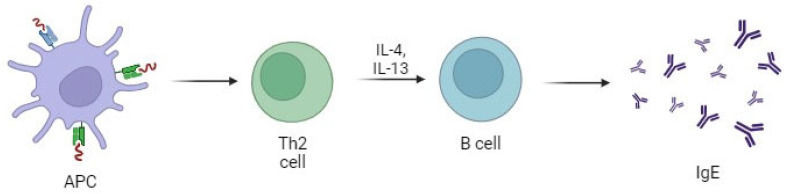
Pathway of IgE Production. Antigen-presenting cells (APCs) process and present antigens to T helper 2 (Th2) cells, which then produce cytokines, such as IL-4 and IL-13. These cytokines stimulate B cells to undergo class-switch recombination and produce IgE antibodies.

IgE is a tetramer formed by two ε-heavy (H) and two light (L) chains (κ or λ), which are linked by several intrachain disulfide bonds. At the N-terminal regions of the heavy and light chains, variable sequences (VH and VL) are present and responsible for antigen-specific binding sites. The C-terminal regions of the heavy chains are made up of four Cε domains (Cε 1–4), and the Cε 2–4 Fc domains confer the IgE isotype-specific functions, including the binding to its receptors (FcεRI and FcεRII) [4]. IgE can be present in two forms: a membrane B-cell receptor form, which is expressed on the surface of B cells that experienced class switching to IgE, and a serum form which is produced by plasma cells [6]. The serum half-life of IgE is 2–3 days, and it is the shortest of all the immunoglobulin isotypes, especially when compared with 23 days for IgG [21]. This short half-life may help minimize IgE cross-reactivity, which can lead to severe anaphylactic reactions. Anaphylaxis is a life-threatening systemic allergic reaction that involves multiple organ systems, often leading to symptoms such as difficulty breathing, a sudden drop in blood pressure, and shock. This indicates that IgE production is tightly regulated, as uncontrolled IgE responses could result in fatal consequences [6,22]. After immunization or infection, activated naïve B cells undergo class-switch recombination (CSR), during which IgE antibodies are generated [23]. Two principal pathways of CSR have been reported: a direct pathway from the IgM to the IgE isotype (direct CSR); and a sequential pathway from the IgM to an IgG1 intermediate and then to IgE (sequential CSR) [24,25]. The Iε promoter regulates transcriptional activation and nucleotide modification that are pivotal for CSR [23,26]. This promoter contains binding sites for signal transducer and activator of transcription (STAT) 6, nuclear factor kB (NFkB), Pax5, E2A, NFIL3, activator protein-1 (AP-1), C/EBP, and PU.1 [4,27,28]. The presence of infectious agents (e.g., parasite infections) or exposure to specific antigens induces the release of damage-associated molecular patterns (DAMPs), alarmins, and cytokines from epithelial cells and innate immune cells, leading to the production of Interleukin (IL)-4 from T helper (T_H_) cells [22,29]. IL-4, by activating STAT6- and NFkB-signaling pathways, induces the ε-germline transcription and the expression of activation-induced cytidine deaminase (AID), which are pivotal for both direct and sequential CSR [9,30]. The NFkB-signaling pathway is activated by the interaction between CD40, expressed on B cells, and its ligand (CD40L), expressed on activated T_H_ cells [31]. The STAT6- and NFkB-binding sites are next to each other and act synergically to drive transcription [32]. The role of STAT6 in the functioning of IL-4 is so crucial that STAT6-deficient mice report many of the alterations observed in IL-4-deficient mice (e.g., perturbations in Th2 differentiation and Ig class switch) [30]. Moreover, it is reported that the targeted inactivation of the p50 subunit of NFkB in mice is associated with a marked decrease in IgE production and in ε-germline transcription, supporting the central role of NFkB as a transcription factor [33,34]. The role of IL-13 in promoting IgE synthesis by the activation of the STAT6-signaling pathway is also described [35]. Interestingly, IL-13 seems to enhance the production of high-affinity IgE from follicular T_H_ (T_FH_) cells, a subset of T_H_ cells localized in the B-cell follicle which express B-cell-stimulating molecules, such as CD40L [36,37] [Figure 1]. Conversely, IL-21 may suppress IgE production through the inhibition of ε-germline transcription by triggering STAT3-dependent signaling [38]. In fact, IL-21 receptor (IL-21R)-deficient mice showed an increased IgE production compared to wild-type animals [39]. The co-expression of all these cytokines (IL-4, IL-13, and IL-21) in T_FH_ cells suggests the presence of an important balance in the regulation of IgE production [39]. TFH cells are crucial not only for IgE production but also for the production of IgG4. While IgE is typically associated with acute and severe allergic reactions, IgG4 plays a more regulatory role, often involved in prolonged and less inflammatory immune responses. Specifically, IgG4 is linked to IgG4-related diseases in adults, which include chronic inflammatory conditions characterized by plasma cell infiltrates. The balance between IgE and IgG4 production, regulated by TFH cells, reflects the modulation of the immune response, with implications for both allergic reactions and autoimmune diseases [36].

### 2.2. Conditions Related to High IgE Levels

**Figure 2:**
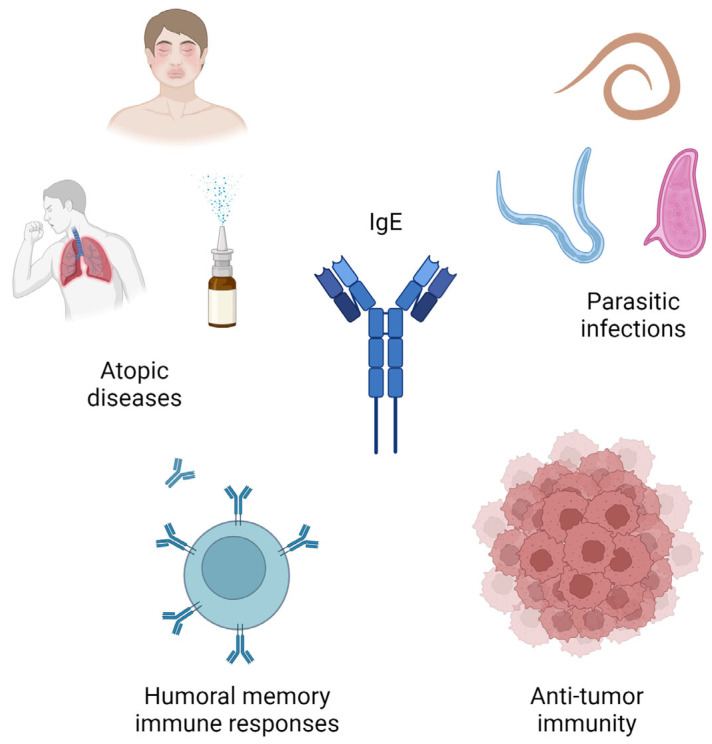
Immunoglobulin E (IgE) plays a crucial role in the immune system, particularly in the context of atopic diseases and defense against parasitic infections. It is responsible for humoral memory immune responses, and it may also be implicated in anti-tumor immunity.

IgE plays a key role in immune defense against parasites, venoms, and toxins [Figure 2]. Parasitic infections can cause a 10 to 100-fold increase in total serum IgE, elevating both parasite-specific and nonspecific IgE levels [40]. These antibodies mediate the expulsion of some forms of parasites by activating Th2 cells and limiting the diffusion of parasitic larvae within eosinophil-rich inflammatory infiltrates [41]. Binding parasite-specific IgE to FcεRI receptors promotes the activation of effector cells, such as mast cells and basophils, which release active mediators favoring parasite expulsion [42]. Another postulated mechanism contributing to parasite expulsion is antibody-dependent cellular cytotoxicity (ADCC) via IgE and/or IgG receptors [43]. Other infections are reported in association with high IgE levels and must be considered in differential diagnosis, such as human immunodeficiency virus (HIV), Epstein–Barr virus (EBV), and candidiasis [44,45,46]. Interestingly, EBV can induce IgE CSR by mimicking CD40 by its virus-encoded latent membrane protein 1 (LMP1) [47]. Increased IgE levels are a hallmark of allergic disorders, and IgE concentrations may reach over 10 times the normal level in atopic individuals [7]. The exposure to specific allergens at epithelial barriers in atopic individuals induces the activation of an innate immune response, with the production of chemokines, cytokines, and alarmins (i.e., IL-1, IL-25, IL-33, and thymic stromal lymphopoietin (TSLP)) and the stimulation of a Th2-mediated immune response. The encounter between the antigen-presenting cells (APCs), such as the DCs, and the naïve CD4 cells stimulates secretions of IL-4 and IL-13 from the Th2 cells, producing specific IgE by the B cells [48,49,50]. The binding of IgE with FcεRI expressed on the mast cells and basophils leads to the release of preform mediators, such as histamine, proteoglycans (heparin), and several proteases (i.e., tryptase, chymase, and carboxypeptidase A3) [51,52]. Histamine induces immediate vasoactive effects that may provoke, in some cases, anaphylaxis and even fatal consequences [53]. Other inflammatory mediators released by mast cells and basophils contribute to the participation of neutrophils and eosinophils in allergic inflammation.

Chronic allergen exposure may induce persistent inflammation, leading to tissue injury and remodeling [49,54]. The role of IgE memory in atopic individuals is currently debated. Interestingly, a recent study showed the presence of a population of type 2-polarized memory B cells (MBC2s) constitutively expressing germline transcripts for IgE. MBC2s may be the primary cells that emerge during the first month of sublingual immunotherapy (SLIT), providing evidence that this population is a reservoir for memory IgE [55]. Moreover, considering the central function of IgE in allergic inflammation, many biological treatments have been commercialized directly against IgE (i.e., omalizumab) or against IgE transcription factors or key cytokines (IL-4 and IL-13) [56,57]. There is also evidence of the implication of IgE in inflammatory and autoimmune diseases, such as systemic lupus erythematosus (SLE), chronic urticaria, and bullous pemphigoid [58]. In SLE, autoreactive IgE, particularly dsDNA-specific IgE, has been identified as a potential marker of disease activity [59]. Elevated IgE levels in SLE patients raise the question of whether IgE contributes directly to the pathogenesis of the disease or if it is simply a byproduct of the underlying autoimmune processes [59,60]. Some studies suggest that dsDNA-specific IgE forms immune complexes that exacerbate tissue damage, enhancing disease activity. Alternatively, elevated IgE might reflect a more general immune dysregulation common in autoimmune diseases, without playing a central pathogenic role [59,60]. Furthermore, the T regulatory (Treg) cell dysfunction observed in these diseases may affect IgE synthesis, explaining the presence of autoreactive IgE [61]. Elevated IgE levels were detected in patients with Kawasaki disease (KD), and those levels were more increased significantly in patients with coronary artery lesions compared to individuals without coronary artery lesions [62]. However, KD could be more frequent in individuals with IEIs due to immune dysregulation, and clinicians should consider this potential association [63]. High serum IgE levels may present in malignant conditions due to secondary B-cell action in cutaneous and/or systemic cancer or to B-cell clonality in hematological conditions (such as IgE myeloma) [64]. However, recent research analyzed the anti-tumor functions of IgE. In a 30-year prospective study in 37,747 individuals, increased levels of serum IgE were associated with a low risk of chronic lymphocytic leukemia without convincing evidence for a high risk of any cancer type [65]. In other studies, elevated IgE levels seem to protect against specific cancer types, such as lung cancer (in the absence of asthma), colorectal cancer, and pancreatic cancer, and prolong the survival of glioma patients [66,67,68,69,70,71]. These results explain the recent development of IgE immunotherapy against cancer, especially for melanoma [72,73]. Further studies are needed to improve our knowledge on this important topic.

## 3. Hyper IgE Syndromes (HIES)

**Figure 3:**
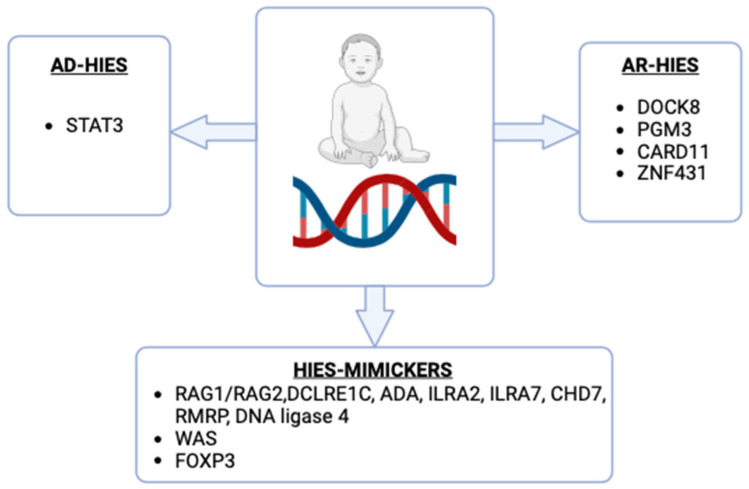
Key genes involved in the pathogenesis of HIES and HIES mimickers. STAT3: Signal Transducer and Activator of Transcription 3; DOCK8: Dedicator of Cytokinesis 8; PGM3: Phosphoglucomutase 3; CARD11: Caspase Recruitment Domain Family Member 11; ZNF431: Zinc Finger Protein 431; RAG1/RAG2: Recombination Activating Genes 1 and 2; DCLRE1C: DNA Cross-link Repair 1C; ADA: Adenosine Deaminase; ILRA2/ILRA7: Interleukin 1 Receptor Antagonist 2 and 7; CHD7: Chromodomain Helicase DNA-binding Protein 7; RMRP: RNA Component of Mitochondrial RNA Processing Endoribonuclease; DNA Ligase 4: Deoxyribonucleic Acid Ligase 4; WAS: Wiskott-Aldrich Syndrome; FOXP3: Forkhead Box P3.

Hyper IgE syndromes (HIES) are a group of primary immunodeficiency disorders characterized by three main features: eczema, recurrent skin and lung infections, and elevated IgE levels [74]. While most cases are sporadic, there are also autosomal-recessive (AR), autosomal-dominant (AD), and X-linked variants [75]. Recent advances in genetic testing have identified specific genetic mutations associated with different forms of HIES [76,77]. AD-HIES is commonly caused by heterozygous mutations in the STAT3 gene, leading to a dominant negative effect, whereas AR-HIES is most often due to biallelic mutations in the DOCK8 gene [78,79]. Additionally, mutations in other genes, such as PGM3, CARD11, and ZNF431, have been linked to HIES, contributing to the genetic diversity of the syndrome [80,81,82,83,84,85] [Figure 3]. The HIES triad is also observed in other primary immunodeficiency and skin disorders, which, despite their phenotypic similarities, require different treatment approaches [86] [Table 1].

### 3.1. Autosomal Dominant-Hyper IgE Syndromes (AD-HIES)

Classic AD-HIES, also known as Job syndrome, is caused by heterozygous mutations in the STAT3 gene, which have a dominant-negative effect [78]. STAT3, a transcription factor involved in the signal transduction of multiple cytokines such as IL-6, IL-10, IL-11, and IL-21, plays a crucial role in the immune system [87]. IL-6 is essential for the differentiation of T-helper-17 (Th17) cells and T follicular helper (Tfh) cells, with Th17 cells being particularly important for defending against extracellular bacteria and fungi [87,88]. STAT3 mutations lead to reduced Th17 cell numbers, contributing to the susceptibility to recurrent bacterial and fungal infections seen in AD-HIES [87]. Characteristic clinical manifestations of AD-HIES include neonatal-onset eczema, recurrent staphylococcal infections, pneumonia, and mucocutaneous candidiasis [89]. A distinctive dermatological feature is a papulopustular rash that resembles neonatal acne within the first two weeks of life [90]. This rash often progresses to eczema and is typically associated with *Staphylococcus aureus*, responding well to anti-staphylococcal antibiotics, which helps differentiate it from other neonatal pustulosis [89]. Eczema in these patients commonly becomes colonized by methicillin-resistant *Staphylococcus aureus* strains, likely due to the high use of antibiotics, which exerts selection pressure on skin bacteria [89]. “Cold” staphylococcal abscesses are a hallmark of STAT3-HIES, frequently recurring even after drainage and requiring prolonged antibiotic therapy [89]. However, similar lesions can also occur in common variable immunodeficiency (CVID), where immunoglobulin levels are significantly reduced or absent. Interestingly, patients with CVID may still exhibit hypersensitivity symptoms despite low or absent IgE levels. This observation raises important questions about the consequences of low IgE in the serum and its role in immune responses. Additionally, further investigation into whether IgE receptors on neutrophils are involved in these severe bacterial infections could provide valuable insights [91]. The predominance of staphylococcal infections in these patients is thought to be associated with an IL-17 deficiency, which may impair the ability of keratinocytes and bronchial epithelial cells to secrete antimicrobial peptides like beta-defensins, thus increasing susceptibility to infections at epithelial sites [86]. Additionally, a recent study by Zhang et al. found that epithelial cells from patients with Job syndrome exhibited a decreased ability to eliminate bacteria and initiate a robust inflammatory response, showing higher adherence of bacteria like *Pseudomonas aeruginosa* and reduced antibacterial activity [92]. This contributes to chronic lung infections and complications such as bronchiectasis and pneumatoceles, commonly seen in patients with AD-HIES [92]. Not only are bacterial infections frequent, but fungal infections are also common due to the involvement of the Th17/IL-17 axis in countering fungal infections [78,93]. Chronic mucocutaneous candidiasis occurs in 70% of patients, typically presenting as oral or genital thrush or onychomycosis [78]. Pulmonary aspergillosis is also common, particularly in patients who develop parenchymal complications [94]. Non-immunological symptoms include skeletal, dental, and connective tissue abnormalities, such as delayed shedding of primary teeth, an increased risk of bone fractures, and distinct facial features [95,96]. The severity of these features may increase with age, complicating early diagnosis. Malignancies occur in about 7% of patients with STAT3-HIES, primarily from hematopoietic tissues, with lymphoma being the most frequently reported [97,98]. Additionally, some STAT3-HIES patients may occasionally present with a lupus-like phenotype, characterized by antinuclear antibodies and anti-double-stranded DNA antibodies [99]. Regarding the risk of allergic disease, patients with STAT3-HIES, despite elevated IgE levels, experience lower rates of allergy and anaphylaxis compared to individuals with similar IgE levels and atopic dermatitis, although these rates are still higher than in the general population [100,101]. This reduced allergic response may be due to impaired mast cell degranulation and the abnormal formation of IgE, which is produced in larger quantities but with lower affinity to allergens [100,101].

Laboratory findings typically show elevated serum IgE levels (ranging from 2000 to 100,000 IU/mL) and eosinophilia, though IgE levels may decrease with age. The laboratory findings generally show elevated serum IgE levels, eosinophilia, and normal immunoglobulin concentrations, although responses to vaccines may be impaired due to abnormal B-cell maturation [89]. The National Institutes of Health (NIH) scoring system remains valid and relevant for diagnosing AD-HIES, through the evaluation of different parameters such as IgE > 1000 IU, characteristic clinical features, lack of Th17 cells, or family history [102]. A very recent study on a large cohort has also highlighted how this score remains current and applicable for population screening [103]. Treatment focuses on meticulous skin care and prophylactic use of antibiotics and antifungals. Early intervention with antimicrobials for skin and lung infections is essential [75]. Although intravenous immunoglobulin therapy is debated, it benefits patients with antibody deficiencies [75]. Despite various controversies in past years, hematopoietic stem cell transplantation (HSCT) appears to remain an effective treatment for the immunological aspects of the disease and also stabilizes severe pulmonary involvement [104]. Recurrent skin infections and abscesses could be eliminated, and donor TH17 cells may produce IL-17A levels comparable to healthy controls [104]. James et al. [105] recently conducted a study on the use of monoclonal antibodies to treat patients with STAT3 dominant negative (STAT3 DN) mutations. The study involved 17 patients receiving different monoclonal therapies targeting allergic and eosinophilic inflammation, including dupilumab, mepolizumab, omalizumab, reslizumab, and benralizumab [105]. The results indicated that dupilumab was particularly effective in treating eczematoid dermatitis, significantly reducing the need for topical treatments and antibiotics [105]. It also showed benefits in managing asthma and allergic bronchopulmonary aspergillosis (ABPA), although the response varied, with some patients experiencing minimal or no improvement [105]. Other monoclonal therapies, such as mepolizumab and omalizumab, provided relief for asthma and ABPA, while reslizumab and benralizumab were effective in reducing eosinophil counts in a patient with eosinophilic esophagitis (EoE), despite the persistence of dysphagia [105]. Overall, the therapies were well tolerated, with only two patients discontinuing treatment due to adverse effects [105].

### 3.2. Autosomal Recessive-Hyper IgE Syndromes (AD-HIES)

#### 3.2.1. DOCK8 Deficiency

DOCK8 deficiency is caused by biallelic mutations in the DOCK8 gene, which plays a key role in cell migration, cytoskeletal rearrangement, and immune synapse formation [106]. DOCK8 is crucial for T-cell and dendritic cell functions, influencing processes such as Th2 skewing, impaired T-cell receptor signaling, and reduced NK cell activity [106]. These deficiencies contribute to a broad range of immune dysfunctions, including combined immunodeficiency, autoimmunity, and atopy [106]. Patients with DOCK8 deficiency manifested early in life as a combined immunodeficiency characterized by eczema, recurrent respiratory infections, and persistent viral skin infections [106]. A hallmark of this condition is a strong predisposition to atopic diseases, including food and environmental allergies, asthma, eosinophilic esophagitis, idiopathic anaphylaxis, and allergic rhinitis, which are less common in AD-HIES [107]. Patients with DOCK8 deficiency are particularly susceptible to severe, persistent, and treatment-resistant cutaneous viral infections, such as *Varicella-Zoster*, *Molluscum Contagiosum*, *Herpes Simplex*, and human *Papillomavirus* (HPV) [108]. These viral infections can also affect deeper tissues and lead to systemic issues, including polymultifocal leukoencephalopathy (PML) caused by the JC virus, and infections involving the central nervous system, eyes, lungs, liver, and gastrointestinal tract [108]. The infectious profile of DOCK8 deficiency also includes recurrent sinopulmonary and cutaneous bacterial infections, especially with *Staphylococcus aureus*, as well as fungal infections ranging from mucocutaneous candidiasis to invasive diseases like aspergillosis and cryptococcosis [106]. Parasitic infections such as *Cryptosporidium*, *Entamoeba histolytica*, and *Giardia lamblia* have also been observed. Autoimmune complications are common in DOCK8 deficiency, including autoimmune hemolytic anemia, chorioretinitis/uveitis, hypothyroidism, cytopenias, and vasculitis [109,110]. Systemic lupus erythematosus (SLE) has been reported in some patients, characterized by purpuric and necrotic skin lesions, arthritis, and glomerulonephritis [111]. DOCK8 deficiency also significantly increases the risk of malignancies, affecting up to 17% of patients [112]. These cancers are often virus-driven, such as HPV-related squamous cell carcinomas and EBV-associated tumors and lymphomas [113]. Non-virally-associated cancers, such as microcystic adnexal carcinoma and aggressive cutaneous T-cell lymphoma, have also been documented [113]. Intestinal complications, including malabsorption and chronic diarrhea, can lead to failure to thrive and growth stunting. These issues may stem from allergic or autoimmune enteropathy or intestinal infections, and, in some cases, can resemble IPEX-like disease [114]. The central nervous system is frequently affected in DOCK8 deficiency, with both infectious (meningitis, encephalitis, abscesses) and noninfectious complications (vasculitis, aneurysms, strokes) being reported [106,115]. Laboratory findings showed eosinophilia, elevated IgE levels, and lymphopenia, particularly affecting T cells. Lymphocyte proliferation is typically reduced, and Ig levels can vary, with low IgM levels being expected and possibly declining with age. Vaccine responses are generally poor [106]. Although patients may score ≥ 40 on the NIH scale, this is less common than in AD-STAT3 deficiency [86]. Flow cytometric analysis for DOCK8 protein expression is a reliable diagnostic tool, as most patients have mutations affecting protein expression [116]. Supportive care, including infection management, prophylactic antimicrobials, and immunoglobulin replacement, is critical. However, without HSCT, the prognosis is poor, with a median survival of around 20–30 years [106]. Unlike STAT3 loss-of-function mutations, the specific impact of DOCK8 deficiency on the immune system makes early HSCT highly successful [117]. In recent years, efforts have been made to identify effective adjunctive therapies. For instance, a study conducted by Ollech et al. [118] concludes that while HSCT remains the gold standard for treating DOCK8 deficiency, dupilumab represents a promising option for managing severe dermatitis in these patients, particularly as a pre-transplant intervention. There was recently reported a case of a seven-year-old girl with DOCK8 deficiency who presented with recurrent wart-like lesions and severe, progressive herpetic infections [119]. The patient benefited from a combined treatment of siltuximab, an IL-6 inhibitor, and prednisone, suggesting that targeting IL-6 with therapies like siltuximab could effectively manage severe infections in patients with DOCK8 deficiency [119].

#### 3.2.2. PGM3, CARD11, and ZNF431

In 2014, AR-HIES caused by homozygous hypomorphic mutations in the phosphoglucomutase-3 (PGM3) gene was identified [80,81]. PGM3 is critical for the synthesis of uridine diphosphate N-acetylglucosamine (UDP-GlcNAc), a precursor necessary for protein glycosylation [120]. While the exact mechanism linking this glycosylation defect to HIES remains unclear, it is hypothesized that UDP-GlcNAc, a substrate for O-GlcNAc transferase, may influence cellular signaling, including TCR signaling via NF-κB and NFAT, leading to Th2 cell expansion [120]. Like other congenital glycosylation disorders, PGM3 deficiency results in a broad range of clinical manifestations beyond immune dysfunction. Patients with PGM3 deficiency exhibit symptoms overlapping with STAT3 and DOCK8 deficiencies. Early-onset atopic dermatitis is common, and while these patients are prone to allergies, food allergies are less prevalent than in DOCK8 deficiency [120]. Recurrent bacterial skin and sinopulmonary infections are typical [80,81]. Viral skin infections and mucocutaneous candidiasis are less frequent than DOCK8 deficiency [80,81]. Additionally, PGM3-deficient patients display facial dysmorphism (wide nostrils, prominent lips) and skeletal abnormalities (scoliosis, joint hypermobility) similar to those seen in STAT3-HIES, though primary teeth retention is not observed [80,81]. Unique to PGM3 deficiency are neurological issues such as developmental delays, intellectual disabilities, epilepsy, and motor symptoms (ataxia, hypotonia). Other clinical manifestations include renal problems, erythema multiforme major, and, in rare cases, a severe combined immunodeficiency-like phenotype [80,81]. Typical laboratory findings in PGM3 deficiency include eosinophilia, elevated serum IgE, B-cell lymphopenia, decreased percentage of memory B cells, and hypergammaglobulinemia. Although antibody responses to protein and carbohydrate antigens are generally protective, T-cell lymphopenia, particularly affecting the CD4+ subset, is common and results in a reversed CD4 ratio [121,122]. Due to significant overlap with AD-HIES, many patients with PGM3 deficiency achieve an NIH score of ≥40, which is higher than what is typically seen in DOCK8 deficiency [86]. Oral supplementation with N-acetyl-galactosamine (GlcNAc) could theoretically bypass the metabolic defect and improve the clinical phenotype [86]. Although clinical data are not yet available to guide this therapy, in vitro studies have shown that GlcNAc supplementation can restore intracellular UDP-GlcNAc levels in PGM3-deficient cells [80,81,86]. Given the hypergammaglobulinemia and normal antibody responses to vaccinations, immunoglobulin replacement therapy may not be beneficial [80,81,86]. There are cases reported in the literature of PGM3 deficiency successfully treated with stem cell transplantation [123,124]. Caspase recruitment domain family member 11 (CARD11) is crucial for T- and B-cell receptor signaling via the NF-κB [125]. Mouse model studies have shown that reduced TCR-NF-κB signaling efficiency differentially affects Treg and Th2 effector cells, leading to allergic symptoms [125]. Heterozygous mutations in CARD11 with a dominant negative effect, allowing for some residual CARD11 activity, have been identified as a cause of severe atopy, recurrent infections, combined immunodeficiency, and elevated IgE levels, thus being classified under HIES [83]. Dorjbal et al. [126] provided a detailed clinical analysis of 44 patients from 18 families carrying 14 confirmed dominant-negative CARD11 mutations. These mutations were found to have high clinical penetrance but showed variable expressivity, indicating a broader range of clinical and immunologic manifestations than previously recognized [126]. Key findings included that, in addition to atopic diseases (89%), the most common symptoms were skin viral infections (68%), mouth ulcers (14%), and lung diseases such as infections, pneumonia, and bronchiectasis (68%) [125]. Other immunologic issues included autoimmunity (20%), neutropenia (14%), hypogammaglobulinemia (11%), and lymphoma (7%) [125]. Urdinez et al. [127] conducted a study on 15 patients with heterozygous CARD11 mutations, revealing significant diversity in clinical presentations, even within the same family. The study highlighted that these mutations could result in either a gain or loss of function, leading to a range of immunological disorders, including severe atopic dermatitis, recurrent infections, and autoimmune issues, including autoimmune hepatitis, neutralizing Factor XI autoantibodies, and nephrotic syndrome [127]. Recently, a pathogenic de novo mutation in the CARD11 gene was identified in a patient with very early-onset inflammatory bowel disease (VEO-IBD), further supporting CARD11 as a potential pathogenic gene in VEO-IBD [127]. The clinical and immunological profiles of patients with CARD11 mutations overlap significantly with those of DOCK8-deficient patients. However, cutaneous viral infections and mucocutaneous candidiasis were less common, and neurological complications were rare, with only one patient experiencing seizures and nystagmus [86,127]. Although IgE levels were elevated in all patients, they were generally lower than in STAT3 deficiency [86,127]. Eosinophilia was consistently present, and immunoglobulin levels were typically normal, although some patients had moderately reduced IgG and elevated IgA levels [86,127]. Vaccine antibody responses varied, with consistently poor responses to carbohydrate antigens. While B- and T-cell counts were normal, anti-CD3 were below normal [86,127]. None of the patients with CARD11 mutations achieved a score of ≥40 on the NIH scoring system [86,127]. The treatment of these patients is truly challenging, and there are currently no standardized guidelines [127]. It involves optimizing atopic dermatitis treatment in most patients and providing immunoglobulin replacement therapy and antibiotic prophylaxis in selected cases [127]. Different immunosuppressive strategies are needed for managing skin and gut inflammation. In some selected cases, HSCT is required, with variable outcomes [127]. A recent study investigated dupilumab and omalizumab’s effectiveness in treating severe atopic symptoms in patients with dominant-negative loss-of-function mutations in the CARD11 gene [128]. The study included three children and three adults, all of whom developed atopic disease in infancy or early childhood, particularly severe atopic dermatitis or chronic spontaneous urticaria, resistant to standard treatments [128]. Dupilumab was used to treat atopic dermatitis in five patients, and omalizumab was used for chronic spontaneous urticaria in one adult [128]. The results showed rapid and sustained improvement in atopic symptoms for all six patients, with no complications during the follow-up period, allowing for a reduction or discontinuation of previous atopic treatments [128]. Recent studies have identified a functional STAT3 deficiency caused by biallelic mutations in the ZNF431 gene, which encodes a transcription factor that binds to specific DNA motifs, including the STAT3 promoter [84,85]. These mutations result in low constitutive levels of STAT3 mRNA, leading to a decrease in Th17 cells, an excess of Th2 cells, and a reduction in memory B cells, closely resembling the immune profile observed in AD-HIES [84,85]. However, ZNF341 deficiency presents a generally milder clinical phenotype than STAT3 deficiency [128]. The most common manifestation is atopic dermatitis, seen in all reported cases, as in STAT3 deficiency [129]. Patients often present with skin abscesses and mucocutaneous candidiasis, although these symptoms are less frequent than in STAT3 deficiency [129]. Respiratory involvement, such as recurrent infections, pneumonia, and bronchiectasis, is also observed but tends to be less severe [129]. Skeletal and connective tissue abnormalities, including facial dysmorphia, scoliosis, joint hyperextensibility, and retention of deciduous teeth, occur less frequently and with less severity than STAT3 deficiency [129]. Notably, vascular abnormalities, common in STAT3 deficiency, have not been observed in ZNF341-deficient patients [129]. Immunologically, ZNF341-deficient patients exhibit profiles like those of STAT3 deficiency, with elevated levels of IgE and eosinophilia. However, unlike STAT3 deficiency, ZNF341-deficient individuals typically display normal inflammatory responses to infections [129]. Immunophenotyping reveals a high frequency of naive CD4+ T cells and low frequencies of central memory T cells, mucosal-associated invariant T cells, memory B cells, and specific innate lymphocyte populations [129]. There is a notable skew toward Th2 cells and a deficiency in Th17 and T-follicular helper cells, contributing to allergic manifestations and increased susceptibility to bacterial and fungal infections. A significant difference from STAT3 deficiency is the frequent observation of low NK cell counts in ZNF341-deficient patients, which is uncommon in STAT3 deficiency [129]. Because extra-immune manifestations were infrequent, only 25% of patients with ZNF431 deficiency achieved an NIH score of ≥40, complicating the diagnosis and underscoring the need for genetic testing for confirmation [85]. Currently, there is no established treatment protocol for these patients. However, due to the prominent immune defects, HSCT may be more effective in managing ZNF431 deficiency than AD-HIES [86,129].

## 4. HIES Mimickers: Disorders with Overlapping Features

In addition to the “classic” HIESs characterized by a common clinical phenotype and related molecular mechanisms, there are several primary immunodeficiency disorders with distinct clinical manifestations and laboratory findings that mimic HIES. These include conditions like Omenn syndrome, WAS, and IPEX [86].

### 4.1. Omenn Syndrome

Omenn syndrome is a rare, severe form of combined immunodeficiency that presents with both autoimmunity and persistent infections. It is characterized by a generalized, thickened, exfoliating rash, alopecia, lymphadenopathy (swollen lymph nodes), and hepatosplenomegaly (enlarged liver and spleen) [130]. The syndrome is often linked to hypomorphic mutations in the RAG1 or RAG2 genes, which lead to partial but defective T and B cell development, but also in mutations in ARTEMIS, encoded by the DNA cross-link repair 1C (DCLRE1C) gene, ADA, ILRA2, ILRA7, CHD7, RMRP, and DNA ligase 4 genes and in association with 22q11 microdeletion syndrome [86,130]. However, new variants have been continuously identified in recent years [131,132,133]. Patients typically exhibit high levels of eosinophils and IgE, with a notable absence of B cells and the presence of highly activated oligoclonal T cells [134]. These T cells are often unable to function properly, leading to severe immune dysregulation [134]. The pathogenesis involves impaired central and peripheral immune tolerance due to defective thymic development, leading to the expansion of autoreactive T cells [134]. While the only curative treatment is HSCT, outcomes for Omenn syndrome are often less favorable compared to other forms of severe combined immunodeficiency (SCID) [113]. However, recent case reports have shown improved outcomes, utilizing a range of donor types, stem cell sources, and conditioning protocols [135,136]. Recent advances in newborn screening and gene therapy promise to improve this challenging condition’s prognosis [137,138,139].

### 4.2. Wiskott-Aldrich Syndrome

WAS is an X-linked inherited immunodeficiency disorder characterized by three primary features: recurrent infections, thrombocytopenia with small platelets, and eczema. WAS is caused by hemizygous mutations in the WAS gene, leading to a deficiency in the WAS protein, which is essential for lymphocyte and platelet development and crucial for regulatory T cell function, cytoskeletal organization, and immune synapse formation [140]. Clinically, thrombocytopenia leads to symptoms like recurrent nosebleeds, prolonged postsurgical bleeding, petechiae, and easy bruising. Patients may experience frequent respiratory infections, severe viral infections (such as herpes simplex and varicella), and invasive fungal infections [141]. Due to dysfunctional Tregs, there is a high incidence of organ-specific autoimmune conditions, including autoimmune cytopenias, inflammatory bowel disease, arthritis, vasculitis, and IgA nephropathy [141]. Additionally, there is an increased risk of malignancies, particularly leukemia, lymphoma, and myelodysplasia [141]. Diagnostic blood tests typically show thrombocytopenia with microthrombocytes, hypereosinophilia, and sometimes anemia [142]. Ig levels are often abnormal, with elevated IgE and IgA, low IgM, and variable IgG. Over time, T-cell counts may decrease, and lymphocyte proliferation and NK cell cytotoxicity are often impaired [142]. Regulatory T cell function is also reduced, and WASp expression is typically low on flow cytometry [142]. Treatment includes prophylactic antibiotics and immunoglobulin replacement to manage immunodeficiency and recurrent infections [141]. Several studies have widely demonstrated that HSCT is a curative treatment for WAS [143,144,145]. However, in recent years, gene therapy has increasingly proven to be an effective curative treatment for patients with severe WAS, especially for those without a suitable donor, providing long-term benefits as well [146,147,148].

### 4.3. Immune Dysregulation, Polyendocrinopathy, Enteropathy, X-Linked (IPEX) Syndrome

IPEX syndrome is a severe, X-linked genetic disorder characterized by immune dysregulation, polyendocrinopathy, and enteropathy [149]. It is caused by FOXP3 gene mutations that lead to dysfunctional Tregs, resulting in a loss of immune tolerance and the subsequent autoimmune manifestations seen in IPEX patients [149]. The genotype– phenotype correlation in IPEX is complex, with the same mutation leading to varying and atypical clinical presentations [149]. The presence of specific FOXP3 isoforms, influenced by mutations, can also affect the disease severity [150]. The syndrome typically presents early in life, with common symptoms including intractable diarrhea due to enteropathy, type 1 diabetes (T1D), and skin disorders such as eczema and dermatitis [150]. Other organ systems can also be affected, including the kidneys, blood, liver, and nervous system, leading to a wide range of complications such as autoimmune hemolytic anemia, membranous nephropathy, and neurodevelopmental issues [150]. The disease’s severity and manifestations can vary greatly among patients, even among those with the same genetic mutation [149]. The diagnosis of IPEX syndrome involves a combination of genetic testing and immunological assessments [149]. Genetic sequencing of the FOXP3 gene is essential for confirming the diagnosis, especially in patients with a clinical history suggestive of IPEX [150]. Immunological tests include flow cytometry to assess the number and function of Tregs, as well as the measurement of autoantibodies and inflammatory cytokines [150]. Additionally, newer diagnostic markers, such as the analysis of TSDR demethylation, a specific epigenetic marker for Tregs, have been introduced to enhance diagnostic accuracy and to monitor disease progression and treatment response [151]. The treatment of IPEX syndrome is challenging and typically involves a combination of pharmacologic immunosuppression and HSCT, but is limited by autoimmune complications, donor availability, and/or graft-vs.-host disease [150]. Barzaghi et al. [152] demonstrated that patients receiving chronic immunosuppressive therapy were affected by disease recurrence or complications, which impacted long-term disease-free survival. When HSCT was performed in patients with a low organ impairment (OI) score, it resulted in disease resolution and improved quality of life, regardless of age, donor source, or conditioning regimen. Gene therapies are increasingly emerging for IPEX syndrome as well. Two primary strategies have emerged: the engineering of CD4+ T cells to express FOXP3, and the gene editing of hematopoietic stem cells (HSPCs) to restore functional FOXP3 expression [153]. The first approach involves the use of lentiviral vectors or CRISPR/Cas9 technology to introduce the FOXP3 gene into CD4+ T cells, effectively converting these cells into Treg-like cells [154]. The second approach focuses on editing the FOXP3 gene directly within HSPCs. Specifically, Singh et al. [155] recently demonstrated that a safer alternative to HSCT could involve correcting the FOXP3 mutation in autologous HSCs through a homology-directed repair (HDR) strategy, utilizing a combination of Cas9 ribonucleoprotein complexes and adeno-associated viral vectors.

## 5. Conclusions

In conclusion, while high IgE levels are commonly associated with atopic diseases, they can also be indicative of IEIs such as HIES, Omenn syndrome, WAS, and IPEX syndrome. This review uniquely integrates recent genetic insights and advances in the understanding of IEIs, emphasizing the importance of considering these conditions in patients presenting with elevated IgE levels, recurrent infections, and autoimmune symptoms. The correct interpretation of IgE levels in conjunction with other clinical and immunological findings is essential to avoid misdiagnosis and to guide the use of advanced diagnostic tools and therapies, such as HSCT and gene therapy. Early diagnosis and tailored treatment can significantly improve the prognosis and quality of life for these patients.

## Figures and Tables

**Table 1:** Key Features of IEIs with Elevated Serum IgE Levels.

Disorder	Gene Mutation	Pathophysiology	Clinical Features	Laboratory Findings	Treatment Options
AD-HIES	STAT3	The mutation affects Th17 cell differentiation, impairing the immune response to extracellular pathogens Reduced IL-6, IL-21, and IL-23 signaling contributes to susceptibility to infections and inflammation.	Neonatal-onset eczema, recurrent staphylococcal infections, pneumonia, mucocutaneous candidiasis, skeletal, dental, and connective tissue abnormalities, malignancies	Elevated IgE levels Eosinophilia Normal to Low Specific Antibody Responses Decreased Th17 Cells Elevated ESR	Skin care Prophylactic use of antibiotics and antifungals Intravenous immunoglobulin therapy HSCT Monoclonal antibodies
AR-HIES (DOCK8 deficiency)	DOCK8	The deficiency disrupts cytoskeletal reorganization in immune cells, causing impaired immune synapse formation, defective T and NK cell migration	Recurrent viral and bacterial infections, eczema, food allergies, autoimmunity, CNS infections	Elevated IgE Levels Eosinophilia Lymphopenia Decreased Th17 Cells Reduced numbers of memory B cells Defective NK Cell Function	Infection Management Prophylactic antimicrobial Ig replacement HSCT Monoclonal Antibodies
AR-HIES (PGM3 deficiency)	PGM3	PGM3 mutations disrupt glycosylation processes, leading to impaired immune cell signaling and function, affecting T, B, and NK cells, resulting in immune dysregulation	Recurrent viral and bacterial infections, skeletal abnormalities, atopy, autoimmunity	Elevated IgE, Lymphopenia	Supportive treatment N-acetylgalactosamine supplementation
AR-HIES (CARD11 deficiency)	CARD11	CARD11 mutations affect T and B cell signaling and activation, causing impaired development of Th2 and Th17 cells	Eczema, recurrent infections, atopy, lymphoproliferation	Elevated igE Abnormal levels of IgG, IgA, and IgM, which can be either low or normal.	Antibiotics Ig replacement
AR-HIES (ZNF431 deficiency)	ZNF431	ZNF431 mutations affect transcriptional regulation of immune-related genes, disrupting T and B cell function and causing immune dysregulation	Recurrent infections, eczema, atopic diseases, developmental delays	Elevated IgE Abnormal or reduced levels of IgG, IgA, IgM. Lymphopenia Decreased Th17 cells	Supportive treatment HSCT
Omenn Syndrome	RAG1, RAG2, and other immune regulatory genes (e.g., DCLRE1, ADA, ILRA2, ILRA7, CHD7, RMRP, and DNA ligase 4)	Mutations impair V(D)J recombination, leading to defective T and B cells, causing immunodeficiency and autoimmunity.	Exfoliating rash, alopecia, lymphadenopathy, hepatosplenomegaly, persistent infections, autoimmunity	Elevated IgE Levels, low IgG, IgA, and IgM Levels Eosinophilia Autoantibodies Absence of B cells. Presence of highly activated, oligoclonal and often unable T cells	HSCT. Gene therapy.
Wiskott- Aldrich Syndrome	WAS gene	WAS mutation disrupts cytoskeletal organization in lymphocytes and platelets, leading to defective T cell and B cell activation, impaired antibody production, and dysfunction in natural killer (NK) cells and platelets.	Recurrent infections, eczema, autoimmune diseases, malignancies	Elevated IgE and IgA levels, low IgM, and variable IgG levels Eeosinophilia Thrombocytopenia with microthrombocytes. Anemia. Reduction of T cell counts and in Tregs function Low WASp expression on flow cytometry	Prophylactic antibiotics Ig replacement HSCT Gene therapy
IPEX Syndrome	FOXP3 gene	FOXP3 mutation causes loss of regulatory T cell function, leading to widespread autoimmune dysregulation.	Intractable diarrhoea, type 1 diabetes, eczema, autoimmune disorders	Elevated IgE Levels Eosinophilia Autoantibodies Hyperglycemia Hypoalbuminemia Thrombocytopenia Anemia Lymphopenia or T-cell Lymphocytosis Reduced or absent FOXP3 expression in Tregs on flow cytometry	Combination of immunosuppressive drugs and HSCT Gene therapy

AD-HIES: Autosomic Dominant Hyper-IgE Syndromes; ADA: Adenosine Deaminase; AR-HIES: Autosomic Recessive Hyper-IgE Syndromes; CARD11: Caspase Recruitment Domain family member 11; CHD7: Chromodomain Helicase DNA-binding protein; DCLRE1C: DNA Cross-Link Repair 1C; DOCK8: Dedicator of Cytokinesis 8; ESR: Erythrocyte Sedimentation Rate; FOXP3: forkhead box P3; HSCT: hematopoietic stem cells transplantation; IEIs: inborn errors of immunity; IgA: Immunoglobulin A; IgE: Immunoglobulin E; IgG: Immunoglobulin G; IgM: Immunoglobulin M; ILRA2: Interleukin Receptor Accessory Protein 2; ILRA7: Interleukin Receptor Accessory Protein 7; IPEX: Immune dysregulation, Polyendocrinopathy, Enteropathy, X-linked; PGM3: Phosphoglucomutase-3; RAG1: Recombination-Activating Gene 1; RAG2: Recombination-Activating Gene 2; RMRP: RNA Component of Mitochondrial RNase P; STAT3: Signal Transducer and Activator of Transcription 3; Th17: T helper 17; Tregs: T regulatory cells; WAS: Wiskott-Aldrich Syndrome; WASp: Wiskott-Aldrich Syndrome protein; ZNF431: Zinc Finger Protein 431.

## Data Availability

No new data were created or analyzed in this study. Data sharing is not applicable to this article.
